# Acute-phase ITIH4 levels distinguish multi-system from single-system Langerhans cell histiocytosis via plasma peptidomics

**DOI:** 10.1186/s12014-015-9089-2

**Published:** 2015-06-18

**Authors:** Ichiro Murakami, Yukiko Oh, Akira Morimoto, Hitoshi Sano, Susumu Kanzaki, Michiko Matsushita, Takeshi Iwasaki, Satoshi Kuwamoto, Masako Kato, Keiko Nagata, Kazuhiko Hayashi, Shinsaku Imashuku, Jean Gogusev, Francis Jaubert, Takashi Oka, Tadashi Yoshino

**Affiliations:** Division of Molecular Pathology, Faculty of Medicine, Tottori University, 86 Nishi-cho, Yonago-shi, Tottori 683-8503 Japan; Department of Pediatrics, Jichi Medical University School of Medicine, Shimotsuke, Tochigi 329-0498 Japan; Division of Pediatrics and Perinatology, Faculty of Medicine, Tottori University, Yonago, Tottori 683-8503 Japan; Department of Pathobiological Science and Technology, School of Health Science, Faculty of Medicine, Tottori University, Yonago, Tottori 683-8503 Japan; Division of Pediatrics and Hematology, Takasago-seibu Hospital, Takasago, Hyogo 676-0812 Japan; Inserm U507 and U1016, Institut Cochin, 75014 Paris, France; AP-HP Hôpital Necker-Enfants Malades, University Paris Descartes (Paris 5), 75006 Paris, France; Department of Pathology, Okayama University Graduate School of Medicine, Dentistry and Pharmaceutical Sciences, Okayama, Okayama 700-8530 Japan

**Keywords:** Inter-alpha-trypsin inhibitor heavy chain 4 (ITIH4) [PDB: Q14624], Langerhans cell histiocytosis, Peptidomics, Interleukin-1 loop model

## Abstract

**Background:**

Langerhans cell histiocytosis (LCH) is a proliferative disorder in which abnormal Langerhans cell (LC)-like cells (LCH cells) intermingle with inflammatory cells. Whether LCH is reactive or neoplastic remains a controversial matter. We recently described Merkel cell polyomavirus (MCPyV) as a possible causative agent of LCH and proposed interleukin-1 loop model: LCH is a reactive disorder with an underlying oncogenic potential and we now propose to test this theory by looking for acute markers of inflammation. We detected MCPyV-DNA in the peripheral blood cells of patients with high-risk organ-type (LCH-risk organ (RO) (+)) but not those with non–high-risk organ-type LCH (LCH-RO (−)); this difference was significant. LCH-RO (−) is further classified by its involvement of either a single organ system (SS-LCH) or multiple organ systems (MS-LCH). In patients with LCH-RO (−), MCPyV-DNA sequences were present in LCH tissues, and significant differences were observed between LCH tissues and control tissues associated with conditions such as dermatopathic lymphadenopathy and reactive lymphoid hyperplasia. Although MCPyV causes subclinical infection in nearly all people and 22 % of healthy adults will harbor MCPyV in their buffy coats, circulating monocytes could serve as MCPyV reservoirs and cause disseminated skin lesions.

**Methods:**

Plasma sample from 12 patients with LCH-RO (−) (5 MS-LCH and 7 SS-LCH) and 5 non-LCH patients were analyzed by peptidomics. Mass spectrometry (MS) spectra were acquired and peptides exhibiting quantitative differences between MS-LCH and SS-LCH patients were targeted.

**Results:**

One new candidate biomarker, m/z 3145 was selected and identified after obtaining a MS/MS fragmentation pattern using liquid chromatography-MS/MS. This peak was identified as a proteolytic fragment derived from inter-alpha-trypsin inhibitor heavy chain 4 (ITIH4, [PDB: Q14624]).

**Conclusions:**

Peptidomics of LCH have revealed that the level of acute-phase ITIH4 distinguishes MS-LCH-RO (−) from SS-LCH-RO (−). Acute-phase proteins serve non-specific, physiological immune functions within the innate immune system. LCH may be a reactive disorder with both underlying neoplastic potential of antigen presenting cells harboring *BRAF* mutations and hyper-immunity of other inflammatory cells against MCPyV infection. Among LCH-RO (−), MCPyV-DNA sequences were present in both MS-LCH tissues and SS-LCH tissues without significant differences. ITIH4 may show that LCH activity or LCH subtypes correlates with the systemic or localized reactions of MCPyV infection.

## Background

LCH is a proliferative disorder in which abnormal Langerhans cell (LC)-like cells (LCH cells) intermingle with inflammatory cells [1, 2]. The appearance of LCH lesions under the microscope, which commonly contain only a small number of abnormal immune LCH cells surrounded by many normal immune cells initially led to questions about LCH as a cancer or immune disorder [3]. For decades, it was thought that the disease is a reactive disorder rather than a neoplastic process [4]. The discovery of the B-Rapidly Accelerated Fibrosarcoma gene (*BRAF*) [GenBank: G_007873] mutation in 2010 [5] gave new insights into LCH pathogenesis. Clonality [6, 7] and *BRAF* mutation [5] suggest neoplasm [1], whereas granuloma formation [8] with spontaneous regression [1], and hypercytokinemia [1, 9] indicate a reactive process. *BRAF* mutation does not distinguish LCH subclasses, which vary from self-healing to lethal [5]. Recently, there has been much discussion concerning whether or not LCH is considered a cancer. This discussion started when a recent change to patient information about LCH on the National Cancer Institute’s website [10] stated, “LCH is a type of cancer that can damage tissue or cause lesions to form in one or more places in the body.” On the contrary, the Histiocyte Society Scientific Committee [3] stated “All cancers are considered neoplasias, but not all neoplasias are cancerous.” At present time no clear consensus seem to have been reached regarding whether LCH is reactive or neoplastic [1] and whether LCH is cancerous [10] or not [3].

Very recently, we [11] propose a new model for LCH pathogenesis in which the disease is a reactive disorder with underlying neoplastic potential. In other words, LCH is an inflammatory process that is prolonged by *BRAF* mutation (interleukin (IL)-1 loop model) [11].

The liver, spleen, and bone marrow are considered high-risk organs for LCH [12, 13]. LCH is classified as involving at least one [LCH-risk organ (RO) (+)] or no high-risk organs [LCH-RO (−)] [12]. LCH-RO (−) presents as multisystem (MS-LCH) or single-system disease (SS-LCH) [14]. Nearly all LCH-RO (+) is MS-LCH-RO (+), although SS-LCH-RO (+) has been reported [15, 16]. We [17] reported Merkel cell polyomavirus (MCPyV)-DNA in the peripheral blood mononuclear cells (PBMC) of patients with LCH-RO (+), whereas MCPyV-DNA was significantly restricted to lesional LCH cells in patients with LCH-RO (−) and so we predict that acute phase markers are related to inflammatory activities of LCH (Table 1) [2]. Berres et al. [18] reported that circulating CD11c + and CD14+ cellular fractions in patients with LCH-RO (+) harbored *BRAF* mutations; the mutation was restricted to LCH cells in patients with LCH-RO (−).

**Table 1:** Clinical manifestations, treatment, outcome and proposed relationship between LCH classification, MCPyV and ITIH4 based on both our and others’ data

Classification			Prevalence (approximate)	Clinical manifestaions	Treatment	Outcome	MCPyV-DNA		Mutations		ITIH4
Present		Former					PBMC	LCH tissue	PBMC	LCH tissue	Plasma
LCH-RO (+)	MS^a^	Letterer–Siwe disease	10 %	Serious anemia, Thrombocytopenia	Multi-agent chemotherapy and Salvage therapy	Mortality rates: 16–38 %	+	NA	+	+	NA
LCH-RO (−)	MS	Hand–Schüller–Christian disease	20 %	Bone pain, Skin rash	Multi-agent chemotherapy	Excellent survival rate	-	+	-	+	high
	SS	Eosinophilic granuloma	70 %	Asymptomatic or Bone pain	Wait-and-see strategy^b^ or Chemotherapy	Excellent prognosis		+		+	low

*ITIH4* inter-alpha-trypsin inhibitor heavy chain 4, *LCH* Langerhans cell histiocytosis, *LCH-RO (+)* LCH with involvement of at least one high-risk organ (spleen, liver, and bone marrow), *LCH-RO (−)* LCH with no involvement of high-risk organ, *MCPyV* Merkel cell polyomavirus, *MS-LCH* multisystem LCH, *SS-LCH* single-system LCH, *NA* not available

^a^Nearly all LCH-risk organ (RO) (+) type is MS-LCH-RO (+), although SS-LCH-RO (+) type has been reported [15, 16]

^b^Localized LCH may resolve spontaneously [2], which might be related to oncogene-induced senescence [45] relayed by interleukin-dependent inflammatory network [46]. We detected MCPyV-DNA in the peripheral blood mononuclear cells (PBMC) of patients with LCH-RO (+) but not those with LCH-RO (−); this difference was significant. In patients with LCH-RO (−), MCPyV-DNA sequences were present in LCH tissues

The clinical course of LCH varies quite widely depending on the extent of organ involvement [2] and relationship between LCH subtypes and MCPyV and mutations are shown roughly in Table 1. Treatment of LCH should be planned according to the clinical presentation and the extent of organ involvement [2]. In LCH-RO (+), in which circulating precursor LCH cells as a reservoirs for MCPyV may relate to involving high risk organ ((extramedullary) hematopoietic organ) (Table 1), main aims of treatment are to increase survive and to reduce the incidence of late sequelae [2]. In LCH-RO (−), treatment differs widely from a wait-and-see approach for SS-LCH to systemic chemotherapy for MS-LCH [2]. However, the clinical significance of MCPyV infection and *BRAF* mutation has not been settled because they occur equally as frequently in MS-LCH-RO (−) and SS-LCH-RO (−) (Table 1) [5].

There are no definite specific laboratory markers for LCH. In many cases, LCH presents with nonspecific inflammatory signs that arise from chronic inflammation [2], although comprehensive analysis of serum levels of cytokines/chemokines and growth factors in pediatric patients with LCH was done [19].

As mentioned above, we predict that acute phase markers are related to inflammatory activities of LCH subtypes (Table 1) and demonstrate, via plasma peptidomics, that acute-phase ITIH4 (inter-alpha-trypsin inhibitor heavy chain 4) levels can distinguish MS-LCH-RO (−) from SS-LCH-RO (−) in this paper.

## Methods

### Patients and plasma samples

This study was approved by the Institutional Review Boards of Okayama University Graduate School of Medicine, Dentistry, and Pharmaceutical Sciences, Okayama, Japan; Faculty of Medicine, Tottori University, Tottori, Japan; and Jichi Medical University School of Medicine, Tochigi, Japan. After obtaining written informed consent, plasma samples were obtained from affected patients in the Japan LCH Study Group registry between 2002 and 2009 and Jichi Medical University School of Medicine.

Plasma sample aliquots from 12 patients with LCH-RO (−) (5 MS-LCH and 7 SS-LCH) were stored at −80 °C until further analysis. For confirmative studies, plasma samples were obtained from 5 non-LCH patients at the Division of Pediatrics and Perinatology, Faculty of Medicine, Tottori University.

### Whole-plasma peptidome analyses (Peptidomics)

The whole-plasma peptidome was directly analyzed via a lithium dodecyl sulfate (LDS)-based 1-D polyacrylamide gel electrophoresis (PAGE)/matrix-assisted laser desorption/ionization (MALDI) mass spectrometry (MS)-based rapid quantitative method using BLOTCHIP® (Protosera, Amagasaki, Japan) [20]: plasma samples were treated with NuPAGE LDS sample buffer (Life Technologies, Carlsbad, CA, USA), heated for 10 min at 70 °C, and applied to NuPAGE Novex Bis-Tris Mini Gels 4–12 % (Life Technologies, No gel is shown). After 1-D PAGE, slab gels were sliced into gel strips; two strips were placed on each chip. Peptides contained in the gel strips were electroblotted onto the chip using an XCell II^TM^ Blot Module (Life Technologies). MALDI matrix-cyano-4-hydroxycinnamic acid (CHCA) (Sigma-Aldrich, MO, USA) was applied to each BLOTCHIP® using an automatic matrix-dispensing machine (Protosera).

MS spectra were acquired on an UltraFlex II MALDI-time of flight (TOF)/TOF (Bruker Daltonics, Billerica, MA, USA) interfaced with flexAnalysis version 2.4 software (Bruker Daltonics) as previously described [20] under the following conditions: laser intensity, 28–37 %; detector voltage, 1685 V; suppression, 500, fuzzy mode; and molecular mass range, m/z 1000–30,000.

All plasma sample measurements were repeated four times. The resulting 29 MS spectra per each measurement were combined using flexAnalysis version 2.4 to generate an integrated MS spectrum in the molecular mass range of m/z 1000–20,000. The mean relative peak intensities, normalized to a total ion current of m/z between 1000 and 6000, were expressed as arbitrary units (a.u.) [21].

### Measurement of monoisotopic masses on BLOTCHIP®

Monoisotopic masses of statistically significant peaks were recorded in reflector mode on an Ultraflex II instrument (Bruker Daltonics).

### Purification and MS/MS identification of peptides

Plasma samples were selected for peptide purification as follows: peptides exhibiting quantitative differences between MS-LCH and SS-LCH patients were targeted based on the statistical analysis of MS data. Blood samples containing the highest target peptide concentrations were used by Protosera as sources for further identification studies.

MS/MS spectra of the target peptides were obtained via liquid chromatography (LC)-MS/MS (Q Exactive, Thermo Fisher Scientific K. K., Yokohama, Japan); fragmentation data were applied to a “nonredundant” human database search (both NCBInr and Swiss-Prot) via the MASCOT MS/MS ions search program version 2.1.0 (Matrix Science, Boston, MA, USA) in Biotools software (Bruker Daltonics).

### Statistical analysis

All statistical MS data analyses were conducted using ClinPro Tools version 2.2 (Bruker Daltonics) [22]. Peak heights exhibiting significant statistical differences between two groups (MS-LCH versus SS-LCH) were selected. MS data intensity comparisons between patients with MS-LCH and SS-LCH were performed using Student’s *t*-test. Differences between values were considered statistically significant at *P* < 0.05.

## Results and discussion

**Fig. 1:**
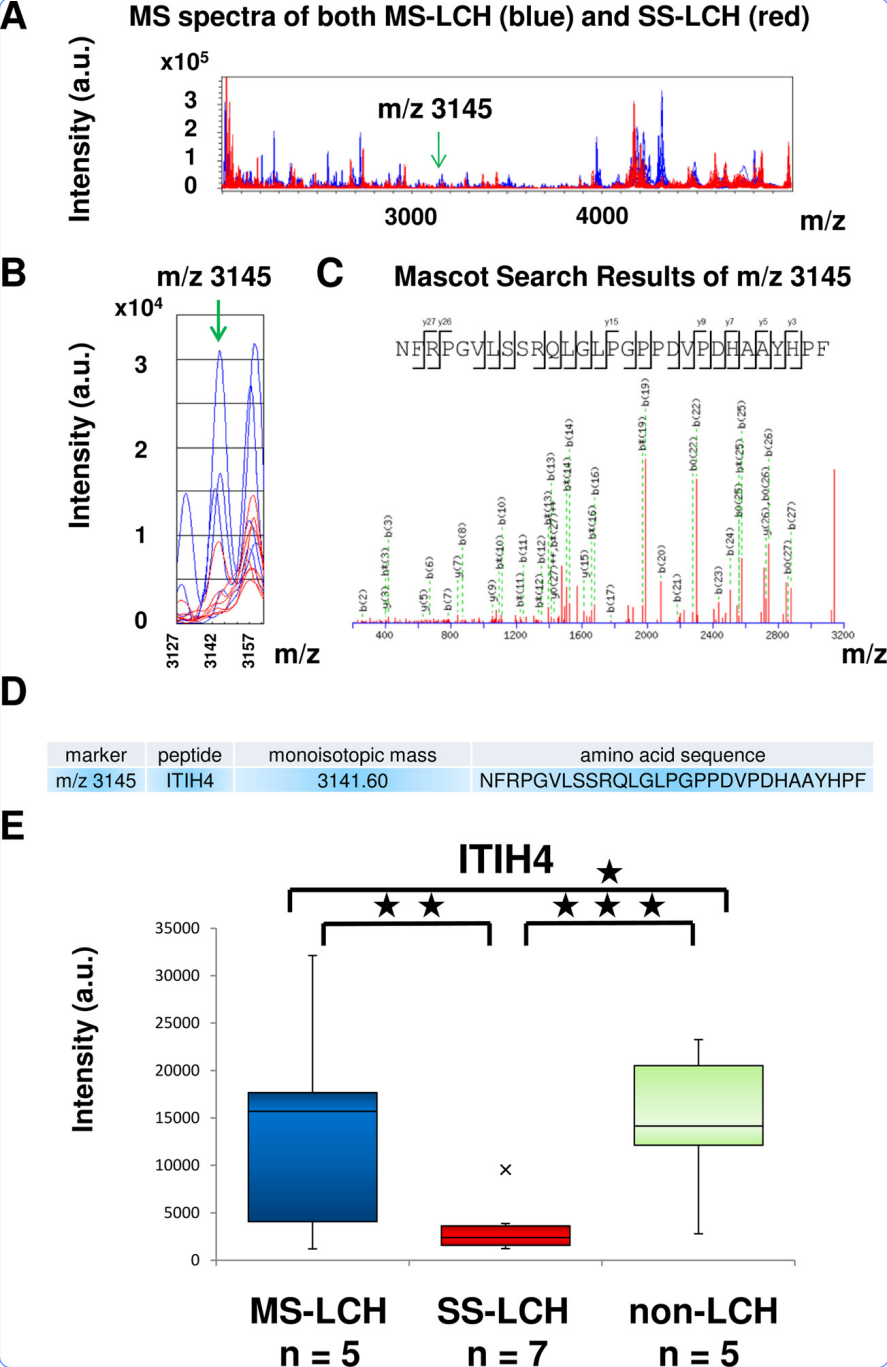
Plasma peptidomics of patients with LCH-RO (−). (**a**) *Blue* and *red lines* indicate integrated MS spectra of each MS-LCH and SS-LCH sample, respectively. All plasma sample measurements were repeated four times. The resulting 29 MS spectra per each measurement were combined using flexAnalysis version 2.4 to generate an integrated MS spectrum. Statistical analysis indicated that 32 peptide peaks differed significantly between MS-LCH and SS-LCH patients at an average m/z of <6000. Peaks (arbitrary unit [a.u.]) in the range of m/z 2000–5000, which contains m/z 3145 (*arrow*), are shown. (**b**) *Blue* and *red lines* indicate each MS spectrum of MS-LCH and SS-LCH samples, respectively. Peaks in the range of m/z 3127–3162, which contains m/z 3145 (*arrow*), are shown. (**c**) Blood samples containing the highest concentrations of the target peptide m/z 3145 were used for further identification studies. MS/MS spectra of the target peptides were obtained via LC-MS/MS (Q Exactive). The MS/MS fragmentation data of m/z 3145 were applied to a “nonredundant” human database search (both NCBInr and Swiss-Prot) using MASCOT MS/MS ions search program version 2.1.0 in Biotools software. MS/MS fragmentations of NFRPGVLSSRQLGLPGPPDVPDHAAYHPF, a peptide found in inter-alpha-trypsin inhibitor heavy chain 4 (ITIH4, [PDB: Q14624]), were detected (ion score: 89; expect: 0.00062). Y-axis: relative intensity. (**d**) A profile summary of peptide fragment m/z 3145 (ITIH4). The amino acid sequence is presented according to the Paris Convention guidelines for peptidomics data presentation. (**e**) Peptidomics data indicating the intensities of m/z 3145 (ITIH4 fragment) in MS-LCH, SS-LCH, and non-LCH plasma samples were plotted as box-whisker plots. For the MS-LCH, SS-LCH, and non-LCH samples, the median intensities are 15710.537, 2412.950, and 14157.62538, lower quartiles are 4081.623, 1583.592, and 12148.52916, and upper quartiles are 17688.457, 3635.496, and 20523.77099, respectively. *P* values (Student’s *t*-test) are also indicated (An *asterisk* means *P* > 0.05. A *double asterisk* means *P* < 0.05. A *triple asterisk* means *P* < 0.01). LCH-RO (−), LCH with no involvement of high-risk organ (spleen, liver, and bone marrow)

Clinical profiles of patients in this study with LCH and non-LCH are summarized in Table 2. Plasma samples from LCH (*n* = 12) and non-LCH patients (*n* = 5) were subjected to BLOTCHIP® followed by MS analysis. Subsequent differential profiling analyses of the two sample sets were performed based on data obtained from the MS analysis. Quantitative differences in peptide peaks between the groups were noticeable primarily in the 2000–6500 m/z range. Statistical analysis indicated that 32 peptide peaks differed significantly between MS-LCH and SS-LCH patients at an average m/z of <6000 (Table 3 and Fig. 1a).

**Table 2:** Clinical characteristics of patients with LCH-RO (−) or non-LCH

Patients	Age	Sex	Diagnosis (subtype)	Distribution of LCH lesions
L1	3 years 0 month	M	MS-LCH	Parietal bone, Ear canal, Lung
L2	7 years 0 month	F	MS-LCH	Bone, Skin, Pituitary gland
L3	4 years 10 months	M	MS-LCH	Skin, Bone, Pituitary gland, CNS
L4	1 year 0 month	M	MS-LCH	Bone, Orbit
L5	11 years 4 months	F	MS-LCH	Bone, Pituitary gland
L6	11 years 11 months	M	SS-LCH	Bone
L7	4 years 11 months	F	SS-LCH	Bone
L8	9 years 0 month	M	SS-LCH	Bone
L9	6 years 0 month	M	SS-LCH	Bone
L10	1 years 0 month	F	SS-LCH	Skin
L11	4 years 4 months	F	SS-LCH	Bone
L12	1 years 3 months	M	SS-LCH	Bone
C1	2 years 4 months	F	Toxicoderma	−
C2	2 years 3 months	M	Kawasaki disease	−
C3	10 years 0 month	F	SLE	−
C4	2 years 3 months	M	ITP	−
C5	1 year 11 months	M	Kawasaki disease	−

The median age of the MS-LCH patients (*n* = 5) was 4 years, 10 months (range: 1 year, 0 months–11 years, 4 months). The median age of the SS-LCH patients (*n* = 7) was 4 years, 11 months (range: 1 year, 0 months–11 years, 11 months). The median age of non-LCH patients (*n* = 5) was 2 years, 3 months (range: 1 year, 3 months–10 years, 0 months). *Abbreviations: CNS* central nervous system, *ITP* idiopathic thrombocytopenic purpura, *LCH* Langerhans cell histiocytosis, *LCH-RO (−)* LCH with no involvement of high-risk organ (spleen, liver, and bone marrow), *MS-LCH* multisystem LCH, *SLE* systemic lupus erythematosus, *SS-LCH* single-system LCH

**Table 3:** Receiver operating characteristic (ROC) analysis of 32 peaks

m/z	SN	SP	Cutoff	AUC
1467	100	57	28,182	0.73
1796	43	100	6693	0.71
1988	100	57	7096	0.78
2013	86	100	8899	0.94
2211	100	43	13,844	0.69
2273	57	100	808	0.84
2555	100	57	18,160	0.67
2601	86	100	2858	0.96
2662^a^	86	43	216	0.63
2727	86	86	3612	0.82
2810	100	43	11,023	0.59
2962^a^	86	57	8557	0.69
3145	86	71	3710	0.73
3159	100	43	14,491	0.71
3291	86	86	1880	0.84
3354	100	57	3537	0.80
3509	100	71	6796	0.84
3522	100	71	4945	0.84
3686	86	86	1603	0.86
3953^a^	71	86	6547	0.76
3973	100	71	15,117	0.82
3990	86	71	6868	0.82
4154	43	100	15,100	0.73
4186	71	86	15,088	0.73
4301	100	71	16,035	0.84
4317	100	100	26,194	1.00
4840^a^	100	57	29,114	0.73
5586^a^	71	71	3706	0.73
7773^a^	100	29	24,831	0.57
8646	71	100	20,684	0.90
15217^a^	86	71	1720	0.80
17492^a^	71	71	8478	0.65

*SN* sensitivity, *SP* specificity, *AUC* area under the curve, *m/z* higher intensity marker in MS-LCH-RO (−), *m/z* ^a^ higher intensity marker in SS-LCH-RO (−)

Three new candidate biomarkers, m/z 2555, m/z 2962 and m/z 3145 (Fig. 1b), differed quantitatively between MS-LCH and SS-LCH patients and were selected. Among three peptides, only m/z 3145 was identified after obtaining a MS/MS fragmentation pattern using LC-MS/MS (Fig. 1c). This peak was identified as a proteolytic fragment derived from ITIH4, ([PDB: Q14624]; Integrated into UniProtKB/Swiss-Prot: July 15, 1998) (Fig. 1d).

ITIH4 is an acute-phase-related protein [23] and potential new biomarker for distinguishing MS-LCH and SS-LCH (Table 3 and Fig. 1e). Acute-phase proteins serve non-specific, physiological immune functions within the innate immune system [24]. ITIH4 has been detected in animals during experimental bacterial and viral infections [25].

MCPyV causes subclinical infection in nearly all people [26]. Although 22 % of healthy adults will harbor MCPyV in their buffy coats [27], circulating monocytes could serve as MCPyV reservoirs and cause disseminated skin lesions [28]. Martel-Jantin et al. [29] reported seroprevalence rate of MCPyV antibodies of children 12 months or younger (49/105) in Cameroon and pointed out the presence of maternal antibodies in very young children. Their data indicated that MCPyV infections mostly occurred during early childhood, after the disappearance of specific maternal antibodies [29]. On the contrary Tolstov et al. [30] reported seroprevalence rate of MCPyV antibodies of children of 1 year or younger (0/6) in patients with LCH. We [17] identified a relationship between LCH and MCPyV. MCPyV-DNA in PBMC correlated with LCH-RO (+) [17]. Among patients with LCH-RO (−) (MS-LCH and SS-LCH), MCPyV-DNA was restricted to lesional LCH cells [17], and we predict that primary MCPyV infection may influence the LCH subtype involving cells in an early-activated state [4].

Generally, no response is observed after secondary viral infection [25]. Primary respiratory syncytial virus infection at 6 months or younger often induces severe disease [31], although nearly all children are infected by 2–3 years of age [32]. Primary Epstein-Barr virus and cytomegalovirus infections in elderly individuals cause a severe condition called infectious mononucleosis; again, nearly all children are infected with these viruses [33]. Although no response is observed after MCPyV infection [26, 27], Kumar et al. [34], however, found that MCPyV-specific T helper cells (in vitro model of a secondary infection) secrete several cytokines, including IL-10. IL-10 is an anti-inflammatory cytokine and is one of cytokines produced in LCH [2, 35]. ITIH4 production is up-regulated by IL-6 [23], which is known produced in LCH [2], one of the mediators coordinating the interface between adaptive and innate immunity [36], and might be a target for the therapy [37]. Innate immune function between newborns and elderly is extremely different and large quantities of IL-6 after stimulation of receptors, such as Toll like receptor, by term newborns are indicated [38]. In LCH, MCPyV infection may induce hyper-immunity in both LCH cells [11] and other inflammatory cells [1, 2]. Increased mRNA expression of *BRAF*, which was proven in MCPyV-positive non-small cell lung cancer [39], may also influence *BRAF* mutated precursor LCH cells.

## Conclusions

LCH is a proliferative disorder in which LCH cells intermingle with inflammatory cells [1, 2]. Very recently, we [11] proposed a new model for LCH pathogenesis in which the disease is a reactive disorder with both underlying neoplastic potential of LCH cells and other inflammatory cells such as T cells, macrophages, and eosinophils. In other words, LCH is an inflammatory process that is prolonged by mutations of antigen presenting cells (IL-1 loop model). The other inflammatory cells may be informed and instigated by the mutated antigen presenting cells. We tested this theory by examining the abundance of a well-established acute phase marker ITIH4. The presence of the acute phase markers observed is compatible with our proposed theory. Cytokine storm is one of characteristics of LCH and the fact that serum levels of some cytokines but not LCH histology, which is so uniform that pathologists cannot determine whether a given biopsy originates from a patient with LCH-RO (+) or LCH-RO (−) [40], deeply correlate to LCH activity and LCH subtypes [19, 41–44] explicitly reach this inflammatory reactive model in LCH.

Peptidomics suggests that the systemic or localized reaction against MCPyV may influence LCH activity or subtypes. Primary infection without maternal immunoglobulins against MCPyV [29, 30] may influence LCH activity or subtypes. In the aspect of data validation, an orthogonal quantitation method has to be conducted, e.g., an ELISA for absolute quantification of ITIH4 in the future.
